# Polygenic risk scores of lithium response and treatment resistance in major depressive disorder

**DOI:** 10.1038/s41398-023-02602-3

**Published:** 2023-09-28

**Authors:** Ying Xiong, Robert Karlsson, Jie Song, Kaarina Kowalec, Christian Rück, Robert Sigström, Lina Jonsson, Caitlin C. Clements, Evelyn Andersson, Julia Boberg, Cathryn M. Lewis, Patrick F. Sullivan, Mikael Landén, Yi Lu

**Affiliations:** 1 Department of Medical Epidemiology and Biostatistics, Karolinska Institutet, Stockholm, Sweden; 2 College of Pharmacy, University of Manitoba, Winnipeg, MB Canada; 3 Department of Clinical Neuroscience, Centre for Psychiatry Research, Karolinska Institutet, Stockholm, Sweden; 4 Stockholm Health Care Services, Region Stockholm, Stockholm, Sweden; 5 Department of Psychiatry and Neurochemistry, Institute of Neuroscience and Physiology, Sahlgrenska Academy, University of Gothenburg, Gothenburg, Sweden; 6 Department of Cognition and Old Age Psychiatry, Region Västra Götaland, Sahlgrenska University Hospital, Gothenburg, Sweden; 7 Department of Psychology, University of Notre Dame, South Bend, IN USA; 8 Social, Genetic and Developmental Psychiatry Centre, King’s College London, London, UK; 9 Department of Medical & Molecular Genetics, King’s College London, London, UK; 10 Departments of Genetics and Psychiatry, University of North Carolina at Chapel Hill, Chapel Hill, NC USA

**Keywords:** Depression, Genomics

## Abstract

Treatment response and resistance in major depressive disorder (MDD) are suggested to be heritable. Due to significant challenges in defining treatment-related phenotypes, our understanding of their genetic bases is limited. This study aimed to derive a stringent definition of treatment resistance and to investigate the genetic overlap between treatment response and resistance in MDD. Using electronic medical records on the use of antidepressants and electroconvulsive therapy (ECT) from Swedish registers, we derived the phenotype of treatment-resistant depression (TRD) and non-TRD within ~4500 individuals with MDD in three Swedish cohorts. Considering antidepressants and lithium are first-line treatment and augmentation used for MDD, respectively, we generated polygenic risk scores (PRS) of antidepressants and lithium response for individuals with MDD and evaluated their associations with treatment resistance by comparing TRD with non-TRD. Among 1778 ECT-treated MDD cases, nearly all (94%) used antidepressants before their first ECT and the vast majority had at least one (84%) or two (61%) antidepressants of adequate duration, suggesting these MDD cases receiving ECT were resistant to antidepressants. We did not observe a significant difference in the mean PRS of antidepressant response between TRD and non-TRD; however, we found that TRD cases had a significantly higher PRS of lithium response compared to non-TRD cases (OR = 1.10–1.12 under various definitions). The results support the evidence of heritable components in treatment-related phenotypes and highlight the overall genetic profile of lithium-sensitivity in TRD. This finding further provides a genetic explanation for lithium efficacy in treating TRD.

## Introduction

Major depressive disorder (MDD) is a leading cause of disability worldwide and is associated with a staggering burden for affected individuals and society [1, 2]. Antidepressants are a first-line treatment for MDD and are generally effective in reducing symptoms and preventing relapse [3]. However, only one-third of individuals with MDD reach complete symptom remission, while another one-third or more fail to respond to antidepressant medications [4]. When individuals with MDD experience an insufficient response to first-line antidepressants, alternative treatment strategies, such as lithium augmentation, switching to a different antidepressant, or a combination of antidepressants, may be used to improve treatment outcomes [5]. Lithium, primarily used to prevent mood episodes in bipolar disorder, has been found to be effective in preventing relapse and hospital readmission in MDD when used as an augmentation therapy [6, 7]. Electroconvulsive therapy (ECT) is typically recommended as second- or third-line treatment for individuals with severe MDD who are not responsive to pharmacotherapy, psychotherapy, or who have an urgent need for rapid clinical improvement in mood due to psychotic symptoms or suicidality [8, 9].

It has been hypothesized that individual variation in the response or resistance to medications used to treat MDD may have a genetic component [4, 10]. For treatment response, the largest genome-wide association study (GWAS) to-date on antidepressant response (*N* = 5218) led by the MDD working group of the Psychiatric Genomics Consortium estimated that 13.2% (95% CI = 2.2–24.2%) of the variance in antidepressant response, measured by symptom remission, was explained by common genetic variants (i.e., SNP-based heritability) [11]. This study also revealed a genetic overlap of antidepressant response with the risk for schizophrenia [11]. Additionally, the International Consortium on Lithium Genetics (ConLi^+^Gen) conducted a GWAS of lithium response in 2563 individuals with bipolar disorder [12]. Although the SNP-based heritability was not reported, this study identified genetic markers associated with a region containing long non-coding RNAs [12]. Follow-up studies also suggested that higher genetic loading for certain psychiatric disorders (MDD and schizophrenia) was associated with poorer lithium response in bipolar disorder, providing evidence for shared genetics between lithium response and risks for psychiatric disorders [13, 14]. The term treatment resistance is used when an individual fails to respond to adequate treatments [15]. Comparing treatment-resistant depression (TRD) and non-TRD cases, the findings from previous studies generally supported that MDD treatment resistance has a genetic component and shares the genetic risks with specific psychiatric disorders [16–19]. For example, a recent study based on UK Biobank with primary care records found that the SNP-based heritability of treatment resistance at 7.7% (95% CI = 2.4–13.0% in the observed scale) and demonstrated that the polygenicity of ADHD was associated with treatment resistance in MDD [17].

Nonetheless, significant challenges remain in defining treatment resistance in psychiatric research [15, 20, 21]. Among MDD, TRD is typically defined by the use of antidepressant medications, and the required number of failed antidepressant trials has been debated [15–18, 20]. The unclear definition of TRD makes it difficult to compare studies and limits our understanding of biological underpinnings in MDD treatment resistance.

In this study, we defined TRD based on the use of both antidepressants and ECT, given that ECT is indicated for individuals with severe MDD who fail to respond to first-line treatment [22, 23]. We also leveraged the latest GWAS of antidepressants and lithium response to investigate the genetic overlap between treatment response and resistance. Specifically, we derived polygenic risk scores (PRS) of antidepressant and lithium response in three Swedish cohorts with over 4500 individuals with MDD, and tested associations of PRS with cases of TRD compared to non-TRD.

## Materials and methods

### Data source

#### Study population

This study consisted of case-only samples with clinical diagnoses of MDD. We extracted MDD cases from three Swedish cohorts. First, we used the Predictors for ECT (PREFECT) study that recruited individuals from the Swedish National Quality Register for ECT between 2013 and 2017 [24]. From the PREFECT study, we extracted the severe MDD cases, i.e., those receiving ECT for a major depressive episode in the context of MDD, but excluding cases receiving ECT for other mood disorders like bipolar or schizoaffective disorder (*N* = 1922) [25]. Second, we used the Internet-based Cognitive Behavior Therapy (iCBT) study which recruited mild-moderate MDD cases who were treated with internet-based cognitive behavior therapy (*N* = 964) [26]. Third, we used the population-based cohort of the Swedish Twin Studies of Adults: Genes and Environment (STAGE), from which we extracted 1686 MDD cases who either had a clinical diagnosis of MDD from the linked patient registers or fulfilled DSM-IV diagnostic criteria, and had no diagnosis of schizophrenia and bipolar disorder [27]. All participants provided informed consent. The studies were approved by the Regional Ethics Review Board in Stockholm. Further details about these samples have been described previously [25–27]. Altogether, 4572 MDD cases were eligible for the study.

#### GWAS summary statistics of antidepressant response

GWAS summary statistics of antidepressant response were obtained from the largest GWAS of antidepressant response to-date (*N* = 5218 MDD cases) [11]. Two phenotypes of antidepressant response were assessed in the GWAS. The first phenotype defined remission as a binary trait, i.e., “whether depressive symptom score in the rating scale decreases to a pre-specified threshold after antidepressant use” (“remission”). The second phenotype was a quantitative trait, defined as “the percentage change of symptoms scale after antidepressant use” (referred to as “percentage improvement”), with a higher percentage improvement indicating a better treatment response. A significant SNP-based heritability was reported only for the binary phenotype “remission” [11], therefore we decided to use the corresponding GWAS summary statistics in the present study. For simplicity and consistency with the previous literature, we referred to this phenotype with the term “antidepressant response” in the remainder of this manuscript.

#### GWAS summary statistics of lithium response

GWAS summary statistics of lithium response were obtained from the largest GWAS on lithium response in patients with bipolar disorder (*N* = 2563) conducted by the ConLi^+^Gen Consortium [12, 28]. Similar to antidepressant response GWAS, two phenotypes of lithium response were assessed: a binary trait dichotomized by a pre-defined cutoff of the rating scale (good vs. poor response to lithium treatment) and a quantitative measure of symptom improvement; to be consistent with the analysis in antidepressant response and MDD treatment resistance, here we also used summary statistics of the binary trait and based on samples with European ancestry (*N* = 2343) in all analyses.

#### GWAS summary statistics of psychiatric disorders

We also obtained GWAS summary statistics of corresponding psychiatric disorders (MDD and bipolar disorder) from the latest published GWAS (*N* = 500,199 in MDD GWAS and *N* = 51, 710 in bipolar disorder GWAS) for sensitivity analyses [29, 30].

### Phenotype definitions

#### TRD

We derived TRD definitions in the PREFECT samples who have received ECT in the context of MDD. Repeated treatment failure is one frequent reason for administering ECT in individuals with MDD [22, 23], but ECT is also used as the first-line treatment in cases of severe psychotic symptoms or life-threatening conditions [31]. Therefore, we utilized the prescription records of antidepressants before the first ECT treatment to ensure our definitions capture actual TRD cases, i.e., individuals with MDD who have received ECT due to treatment resistance. The drug prescription records were obtained from the Swedish Prescribed Drug Register (PDR, available between July 2005 to May 2018) [25], using Anatomical Therapeutic Chemical (ATC) codes under the category of “N06A” for antidepressants and N05AN01 for lithium. We defined TRD cases in the PREFECT samples as those who have used at least one (“*narrow_1*” TRD definition) or two different antidepressants (“*narrow_2*” TRD) of adequate duration before the first ECT treatment. The treatment duration for each antidepressant was calculated based on the first and last dispense date of the antidepressant in the same treatment period (i.e., the time interval between two consecutive prescriptions for the same antidepressant within 120 days [32]). Similar to other studies, we considered adequate treatment duration of ≥6 consecutive weeks in order to account for the expected length of therapeutic effect and to distinguish from drug switches due to adverse effects [17, 33]. For comparison, we also considered a “*broad* definition” without restriction for antidepressant use before the first treatment of ECT.

#### Non-TRD

The comparison group of non-TRD cases was derived from the iCBT and STAGE samples. To match the broad definition of TRD, we considered those without ECT as non-TRD. Since the iCBT samples were mild-moderate MDD cases recruited to begin internet-based cognitive behavior therapy [26], these samples were unlikely to be treated with ECT. For the STAGE samples, we obtained medication data from the PDR to define adequate antidepressant duration similar to our definition in TRD. These STAGE samples were also linked with the patient register from which we identified 19 individuals who had received ECT. In STAGE, non-TRD cases were defined as MDD cases who had used antidepressants but with no more than two antidepressants of adequate duration (≥6 weeks) and had never used ECT. This definition was chosen to capture likely antidepressant responders and to correspond with the commonly used cut-off in the literature for defining TRD/non-TRD cases [20].

Taken together, we derived three sets of comparisons, with one broad definition based on ECT use only and two narrow definitions integrating both ECT and antidepressant use:

1. *Broad*: MDD cases receiving ECT treatment (TRD), compared with those without ECT (non-TRD);
2. *Narrow_1*: MDD cases with ≥1 antidepressant of adequate duration before the first ECT (TRD), compared with those with ≤2 antidepressants of adequate duration and without ECT (non-TRD);
3. *Narrow_2*: MDD cases with ≥2 different antidepressants of adequate duration before the first ECT (TRD), compared with those with ≤2 antidepressants of adequate duration and without ECT (non-TRD).

### Genotyping, quality control, and imputation

Genotyping was conducted at Life and Brain GmbH (Bonn, Germany) for both PREFECT and iCBT samples, using Illumina Infinium Global Screening Arrays (v1) [25, 26]. Samples from STAGE were genotyped with the same array by the SNP&SEQ Technology Platform (Uppsala, Sweden) [27].

After harmonizing the markers and allele coding, we merged raw genotype data from the three studies. Before merging, we excluded SNPs from each study for the following reasons: monomorphic sites; indels; strand ambiguous; and minor allele frequency (MAF) < 0.01. In the merged data, 4187 MDD cases had both genotypes and phenotypes. We then used the PGC Ricopili pipeline for quality control (QC) [34]. After first removing SNPs with missingness >5%, we removed 14 samples (0.33%) due to any of the following criteria: per-sample call rate <0.98; excessive heterozygosity (FHET outside ±0.20); or sex mismatch. We excluded SNPs due to any of the following: per-SNP call rate <0.98; invariant; Hardy–Weinberg disequilibrium (*P* < 1e-06 in controls and cases separately); difference in call rate between cases and controls >0.01; MAF < 0.01. We retained 459,906 SNPs (92.11%) after QC. By projecting the first two principal components (PCs) of the study samples to the reference panel of the 1000 Genome global population, we identified and excluded 56 (1.34%) non-European ancestral outliers whose first two PCs exceeded six standard deviations from the mean values of the European samples in the reference population. Relatedness was estimated from the genotype data and one in each pair of related individuals (75 pairs with $\hat{\pi }\,$> 0.2; $\hat{\pi }$ is the estimated proportion of the genome shared identical-by-descent) was excluded.

After QC, the Sanger imputation service was used to impute genotype data to the reference panel of Haplotype Reference Consortium data (HRC1.1). EAGLE2 and IMPUTE2 were used for pre-phasing and imputation, respectively [35–37].

### Statistical analyses

#### Polygenic risk scores (PRS)

Before calculating the PRS, we further excluded the following SNPs from the GWAS summary statistics: (1) SNPs with MAF < 0.1 or INFO score < 0.9; (2) duplicate SNPs; (3) strand-ambiguous SNPs; (4) SNPs in the major histocompatibility complex regions (chr6:28–34 Mb). SNPs overlapping with Hapmap3 were extracted, and the summary statistics were rescaled to account for linkage disequilibrium (LD) using SBayesR, which is a state-of-the-art method with high prediction accuracy in psychiatric disorders [38, 39]. Finally, the PRS was calculated in the imputed data for each individual as the sum of the number of risk alleles weighted by allelic effects in PLINK (version 2.0) [40]; and was standardized within the whole sample. We also calculated the PRS of MDD and bipolar disorder in the same way for sensitivity analyses.

#### Association analysis

To examine the genetic association of treatment response with TRD status, we first tested the mean differences in the PRS of antidepressant or lithium response among TRD cases compared to non-TRD cases (*t*-test). Logistic regression was used to estimate the odds ratios (OR) corresponding to a per standard deviation (SD) increase in the PRS of antidepressant or lithium response, adjusting for the first four principal components (PCs). We ran these models in all phenotype comparisons. The proportion of the variance in MDD treatment resistance explained by the PRS (Nagelkerke’s *R*^2^) was calculated by comparing the full model, including both PRS and covariates, to the baseline model, which only included covariates. We converted Nagelkerke’s *R*^2^ to the liability scale by assuming that 10% of MDD cases met our stringent definition of TRD. We tested the trend of association in PRS quartiles with treatment resistance using the Chi-squared test.

We applied a false discovery rate (FDR) to control for multiple testing (FDR < 0.05 for six tests—two PRS of treatment response in three sets of TRD/non-TRD comparisons). All analyses were conducted in R (Version 4.0.0) [41].

#### Sensitivity analysis

To account for potential genetic overlap between treatment response and corresponding psychiatric disorder risk (MDD and bipolar disorder) [13, 42], we additionally adjusted for the PRS of MDD and bipolar disorder in the main models.

To examine whether the results were driven by lithium use in TRD patients, we further excluded patients with lithium use and estimated the association between the PRS of lithium response and treatment resistance in MDD.

## Results

After QC, 1778 MDD cases who had received ECT and 2264 without ECT were available for analyses. Demographic and clinical characteristics of samples are shown in Table S1. We further utilized the prescription records of antidepressants before the first ECT treatment to define TRD. Among the MDD cases with ECT (“*broad TRD*” definition), 1674 cases (94.2%) had used antidepressants before the first ECT. The vast majority of these cases—1487 (83.6%) and 1081 (60.8%), respectively—had at least one or two different antidepressants of adequate duration (“*narrow_1 TRD*” and “*narrow_2 TRD*”), suggesting that these MDD cases receiving ECT were resistant to antidepressants. Among the 2264 MDD cases without ECT (“*broad non-TRD*”), 1483 (66.5%) had no more than two antidepressants of adequate duration (“*narrow non-TRD*”) (Table 1).

**Table 1:** Sample size and mean differences in PRS of antidepressant and lithium response.

Definition	Sample sizes		Antidepressants response			Lithium response		
	TRD	non-TRD	Mean difference in PRS_standardized_	*P*	*P*_FDR_	Mean difference in PRS_standardized_	*P*	*P*_FDR_
Broad	1778	2264	−0.015	0.631	0.794	0.094	0.003	0.012*
Narrow_1	1487	1483	−0.010	0.794	0.794	0.107	0.004	0.012*
Narrow_2	1081	1483	0.013	0.742	0.794	0.104	0.009	0.018*

*FDR < 0.05.

To investigate the genetic overlap between treatment response and resistance in MDD, we calculated the PRS of antidepressants and lithium response and evaluated their associations with treatment resistance by comparing the PRS burdens among TRD and non-TRD cases.

With the polygenic component underlying antidepressant response [11], we would expect an inverse association between the PRS of antidepressant response and treatment resistance in MDD. For the *broad* and *narrow_1* definitions, the estimates were in the expected effect direction, i.e., TRD cases had a slightly lower PRS of antidepressant response compared with non-TRD cases, but the mean difference was not significant (Table 1). We found similar but non-significant results in the association analysis adjusting for the population stratification (e.g., OR = 0.98, 95% CI = 0.92–1.06, *P* = 0.672, *P*_FDR_ = 0.775 under the *narrow_1* definition; Table S2). The results remained unchanged after further adjusting for the PRS of MDD (Table S2).

**Fig. 1:**
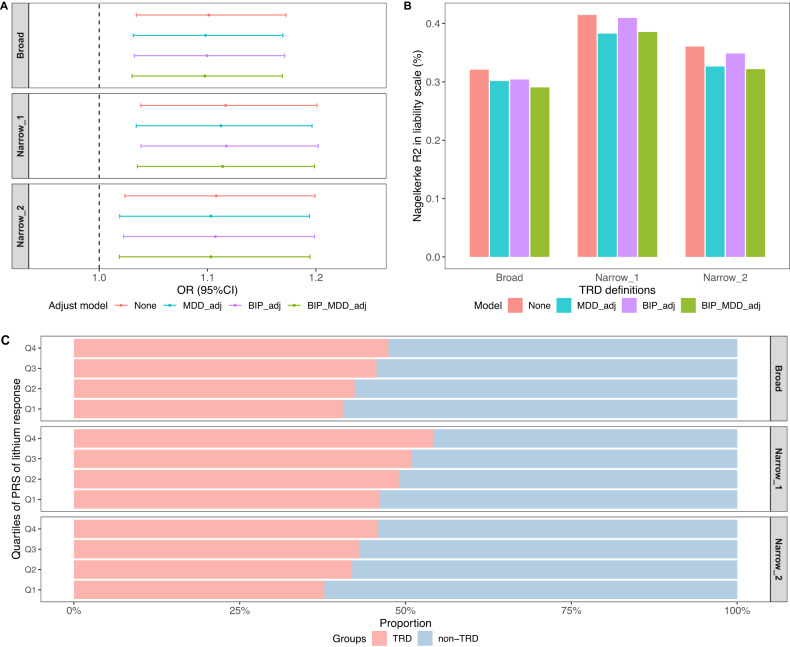
The results of lithium response PRS and TRD. **A** The association between PRS of lithium response and TRD. ORs for TRD associated with per-SD increase in the PRS of lithium response. Each panel shows ORs under different TRD definitions (see the “Materials and methods” section). **B** The proportion of variance in MDD treatment resistance explained by PRS of lithium response (Nagelkerke’s *R*^2^ on the liability scale). **C** The proportion of MDD cases (TRD and non-TRD) in each quartile (Q1–Q4) of the PRS of lithium response. Each panel represents one definition of TRD and non-TRD (see the “Materials and methods” section). The *P* value for trend analysis was 0.0006, 0.0015, and 0.0036 under *broad*, *narrow_1*, and *narrow_2* definitions, respectively. “None”: model adjusted only for the first four PCs. | “MDD_adj”: model additionally adjusted for the PRS of MDD. | “BIP_adj”: model additionally adjusted for the PRS of bipolar disorder. | “BIP_MDD_adj”: model additionally adjusted for the PRS of both MDD and BIP.

Given that lithium is recommended as an augmentation therapy for MDD patients who have experienced insufficient response to first-line antidepressants, we further tested the association of the lithium response PRS with MDD treatment resistance. TRD cases had significantly higher PRS of lithium response than non-TRD cases (mean difference = 0.11, *P* = 0.004, *P*_FDR_ = 0.012 under *narrow_1* definition; Table 1). We found significant associations between PRS of lithium response and TRD in the logistic regression model across all definitions, with per SD increase in the lithium response PRS associated with OR = 1.12 (95% CI = 1.04–1.20, *P* = 0.003, *P*_FDR_ = 0.009 under the *narrow_*1 definition; Fig. 1A, Table S2), although the variance explained was small (Fig. 1B). Further adjustment for additional PRS of MDD, bipolar disorder, or both, did not change the estimates. Similar results were observed after excluding TRD cases with lithium use (*N* = 502, 454, and 357 under *broad*, *narrow_1* and *narrow_2* definitions), suggesting that the association was not due to the effect of lithium use among TRD cases (Table S3). To further quantify the effect of a polygenic load of lithium response among MDD, we divided the MDD cases into quartiles based on their PRS of lithium response and observed a clear trend of a higher proportion of TRD cases in the higher PRS quartiles (*P*_trend_ < 0.005 in all definitions; Fig. 1C). Taken together, the results suggested that TRD cases have a higher polygenic load of responding to lithium compared to the non-TRD cases.

## Discussion

Leveraging the unique resource of clinically ascertained cohorts with comprehensive treatment records and the latest GWAS of treatment response, we derived the definition of TRD and non-TRD and further investigated whether polygenic scores of treatment response differ between TRD and non-TRD cases. We did not observe a significant difference in PRS of antidepressant response between TRD and non-TRD, but we found that compared to non-TRD, patients with TRD had a significantly higher genetic load of lithium response. These results provide evidence for the genetic overlap of treatment response and resistance in MDD and reveal *an overall genetic profile of lithium-sensitivity in patients with TRD*.

To the best of our knowledge, this is the first study using the detailed treatment records of both antidepressants and ECT to derive definitions of TRD and non-TRD. Recent reviews highlighted the key outstanding issue in the research of psychiatric treatment resistance regarding its definition [15, 20, 21]. Indeed, three core components—correct diagnosis, adequate treatment, and non-response—are required to establish treatment resistance [15]. Data from clinical trials are useful for this purpose; however, there is a paucity of clinical trials with sufficient size and adequate length of follow-up. Recently, emerging efforts utilizing Electronic Health Records provide an exciting opportunity to study treatment response and resistance in large-scale, real-world healthcare settings [17, 43]. Using comprehensive clinical records from the Swedish healthcare registers, our work extends these efforts and further demonstrates that integrating the information on ECT use can provide a useful TRD definition. ECT is used to treat individuals with TRD [22, 23], and we also used antidepressant prescription records to confirm that these individuals had multiple trials of adequate antidepressant drugs prior to ECT. Notably, our TRD definitions based on ECT and antidepressant use are more stringent and likely to ascertain more severe patients with TRD compared to those fulfill common TRD definitions based on failed antidepressant trials. One key advantage of using a stringent definition is to minimize misclassification between TRD and non-TRD groups which is common in the categorical-based definitions with certain cutoffs [44]. In addition, ascertaining severe TRD samples can be useful, particularly for research purposes, e.g., extreme phenotyping has been shown as a powerful strategy for novel genetic discoveries [25, 45]. Finally, our definitions fit better with the recently proposed concept of “difficult-to-treat depression” (DTD). The DTD concept differs from the conventional TRD concept in that it moves away from the exclusive focus on acute symptomatic response and instead aims to reduce the depression burden despite usual treatment efforts [21, 46, 47].

Our work extended previous genetic studies of treatment response and further demonstrated the genetic overlap between treatment response and resistance in MDD. We did not find a significant difference in the PRS between TRD and non-TRD cases, but the effect appeared in the expected direction. Similar results were found in the Generation Scotland study (177 TRD vs. 2455 non-TRD; expected effect direction but also non-significant) using TRD definition with at least two switches between antidepressants [11], suggesting that our stringent definitions yielded consistent findings as other definitions commonly used in the literature. The non-significant result may reflect the heterogeneous phenotype of antidepressant responses and limited power from both the GWAS of antidepressant response and the target sample. Nonetheless, the result should be interpreted with caution. Further studies with better power and including the comparisons across different TRD definitions are warranted.

Lithium is known to be effective as an augmentation for patients with TRD [5, 48]. Our novel finding based on PRS, where TRD cases had notably higher genetic load to respond to lithium than non-TRD cases, adds to the evidence of lithium efficacy in treating TRD and further demonstrates that its effect is underpinned by genetic mechanisms. The mechanisms of action of lithium are unclear; however, earlier hypotheses suggested that it plays a role in 5-HT neurotransmission and SNPs in the 5-HT transporter gene have been correlated with response to lithium in patients with depression [13, 49–52]. If the results are replicated, this finding will motivate future genetic studies to reveal the mechanisms of actions involved in lithium.

This study features a stringent measure of MDD treatment resistance, along with the use of the largest GWAS to-date of treatment responses and unique clinical cohorts to investigate the genetics of treatment response and resistance in MDD. In spite of this, the sample size of the GWAS on antidepressant and lithium response is relatively small compared to the GWAS on disease risks. With the continued effort of large consortia to increase sample sizes in these treatment response studies; further validation of our findings is needed. Another limitation of our study is that we used the GWAS of lithium response within bipolar disorders, instead of the GWAS of lithium response within MDD, to investigate the shared genetics between lithium response and MDD treatment resistance. To date, there has been no GWAS study on lithium response within MDD. However, based on previous research, we expect similar mechanisms of lithium response in these two patient groups [53–55]. Nevertheless, future studies are warranted. Furthermore, we examined the response to antidepressants and lithium, but could not evaluate antipsychotics response here. Similar to lithium, atypical antipsychotics are also recommended as augmentations in MDD patients non-responding to antidepressants [56]. Previous GWAS have been conducted on antipsychotic response or resistance, although few specifically targeted the atypical antipsychotics used in MDD and with a sufficient sample size [57, 58]. However, given that the effect of antipsychotic augmentation in TRD is similar to that of lithium [59], we might expect similar findings for antipsychotic response. In addition, we lacked data on psychotherapies and other non-pharmacotherapies to integrate them into our TRD definition. This is a common issue when defining treatment resistance in psychiatric disorders [21]. However given that psychotherapy is used as a first-line treatment (before or combined with antidepressant use), it is less likely to impact our stringent TRD definition based on ECT use. Further efforts to incorporate multiple therapies are needed to refine the treatment resistance phenotype [9]. Meanwhile, our study was conducted with individuals of European ancestry, and future research including other ancestry backgrounds is needed to generalize our findings.

In summary, we derived a stringent TRD definition based on antidepressant and ECT use to capture severe MDD with treatment resistance. Our results highlighted that patients with TRD have a significantly higher genetic load of lithium response compared to non-TRD. This finding forms a genetic explanation for the effectiveness of lithium in treating TRD patients.

### Supplementary information

Supplements

## Data Availability

R scripts used for phenotype derivation and statistical analysis can be shared upon request.
