# Supplementary material for: Polygenic risk scores of lithium response and treatment resistance in major depressive disorder
Source: Transl Psychiatry. 2023 Sep 28;13:301. doi: 10.1038/s41398-023-02602-3 (PMC10539379; doi:10.1038/s41398-023-02602-3)
Supplement: Supplementary file 1 — Supplements [file 41398_2023_2602_MOESM1_ESM.docx]

*Supplementary*

**Polygenic risk scores of lithium response and treatment resistance in major depressive disorder**

Xiong *et al.*

Table S1. Demographic and clinical characteristics of samples.

Table S2. Association between polygenic risk scores (PRS) of antidepressants/lithium response and treatment-resistant depression (TRD).

Table S3. Association between PRS of antidepressants/lithium response and TRD, excluding TRD cases with lithium use.

**Table S1. Demographic and clinical characteristics of samples.**

|  | PREFECT (TRD) | | | STAGE (non-TRD) | | | iCBT (non-TRD) |
| --- | --- | --- | --- | --- | --- | --- | --- |
|  | Broad (N=1778) | Narrow_1 (N=1487) | Narrow_2 (N=1081) | Broad  (N=1362) | Narrow_1  (N=581) | Narrow_2  (N=581) | Broad \| Narrow_1 \|Narrow_2  (N=902) |
| Age^a^ | 54.4 (SD=16.7) | 54.1 (SD=16.7) | 53.4 (SD=16.4) | 45.5 (SD=7.8) | 45.9 (SD=7.6) | | 37.9 (SD=11.8)^b^ |
| Sex (% female) | 1078 (60.7%) | 924 (62.1%) | 685 (63.4%) | 1007 (73.9%) | 448 (77.1%) | | 586 (65.5%) |
| MDD main diagnosis or fulfill DSM-IV criteria | | | | | | | |
| fulfil DSM-IV  (only for STAGE) | - | - | - | 1035 (76.0%) | 371 (63.9%) | | - |
| No. MDD main diagnosis  median (IQR) | 3 (2-5) | 4(2-5) | 4(3-5) | 1 (1-2) | 1 (1-2) | | ^n/a^ |
| mild (F320/F330) | 348 (19.6%) | 313 (21.0%) | 243 (22.5%) | 58 (17.7%) | 36 (17.1%) | | 304 (33.7%)^c^ |
| moderate (F321/F331) | 1089 (61.2%) | 988 (66.4%) | 764 (70.7%) | 107 (32.7%) | 65 (31.0%) | | 509 (56.4%)^c^ |
| severe (F322/F332) | 1240 (69.7%) | 1078 (72.5%) | 803 (74.3%) | 43 (13.1%) | 25 (11.9%) | | 72 (8.0%)^c^ |
| severe with psychotic features (F323/F333) | 488 (27.4%) | 367 (24.7%) | 231 (21.4%) | 4 (1.2%) | 2 (1.0%) | | ^n/a^ |
| Other (F324/F325/F328/ F329/  F334/F335/F338/F339) | 96 (5.4%) | 81 (5.4%) | 58 (5.4%) | 144 (44.0%) | 86 (41.0%) | | ^n/a^ |
| Medications | | | | | | | |
| Ever used antidepressant | 1769 (99.5%) | 1487 (100.0%) | 1081 (100.0%) | 711 (52.2%) | 581 (100%) | | ^n/a^ |
| No. Antidepressant use with adequate duration (> 6 weeks)  median (IQR) | 2 (1-3) | 2 (1-4) | 3 (2-4) | 1 (1-2) | 1 (1-2) | | ^n/a^ |
| Ever used lithium | 502 (28.2%) | 454 (30.5%) | 357 (33.0%) | 4 (0.3%) | 4 (0.7%) | | ^n/a^ |
| Ever used atypical antipsychotic^d^ | 1262 (71.0%) | 1085 (73.0%) | 837 (77.4%) | 45 (3.3%) | 13(2.2%) | | ^n/a^ |

^n/a^ not available.

^a^Age at the end of the follow-up (2016-12-31)

^b^In iCBT it is mean age at inclusion

^c^In iCBT it is MDD diagnosis at inclusion

^d^Atypical antipsychotics used in TRD according to Swedish guidelines: aripiprazole, olanzapine, risperidone, and quetiapine

**Table S2.** **Association between polygenic risk scores (PRS) of antidepressants/lithium response and treatment-resistant depression (TRD).**

| **PRS** | **Definition** | **Main** | | **Additionally adjust PRS of MDD** | | **Additionally adjust PRS of BIP** | | **Additionally adjust PRS of MDD & BIP** | |
| --- | --- | --- | --- | --- | --- | --- | --- | --- | --- |
|  |  | **OR (95%CI)** | **P (***P_FDR_*) | **OR (95%CI)** | **P (***P_FDR_*) | **OR (95%CI)** | **P (***P_FDR_*) | **OR (95%CI)** | **P (***P_FDR_*) |
| AD response | Broad | 0.98  (0.92-1.04) | 0.544  (0.775) | 0.99  (0.93-1.05) | 0.663  (0.789) |  |  |  |  |
| AD response | Narrow_1 | 0.98  (0.92-1.06) | 0.672 (0.775) | 0.99  (0.92-1.06) | 0.789  (0.789) |  |  |  |  |
| AD response | Narrow_2 | 1.01  (0.93-1.10) | 0.775 (0.775) | 1.02  (0.94-1.10) | 0.669  (0.789) |  |  |  |  |
| Lithium response | Broad | 1.10  (1.03-1.17) | 0.003  (0.009)* | 1.10  (1.03-1.17) | 0.003 (0.012)* | 1.10  (1.03-1.17) | 0.003 (0.009)* | 1.10  (1.03-1.17) | 0.004 (0.012)* |
| Lithium response | Narrow_1 | 1.12  (1.04-1.20) | 0.003 (0.009)* | 1.11  (1.03-1.20) | 0.004 (0.012)* | 1.12  (1.04-1.20) | 0.003 (0.009)* | 1.11  (1.04-1.20) | 0.004 (0.012)* |
| Lithium response | Narrow_2 | 1.11  (1.02-1.20) | 0.011 (0.022)* | 1.10  (1.02-1.19) | 0.015 (0.030)* | 1.11  (1.02-1.20) | 0.012 (0.024)* | 1.10  (1.02-1.19) | 0.016 (0.032)* |

PRS: polygenic risk score; BIP: bipolar disorder; MDD: major depressive disorder; AD: antidepressants.

* FDR<0.05 after correcting for multiple comparisons.

All models were adjusted for the first four PCs.

PRS of BIP or PRS of MDD+PRS of BIP were only additionally adjusted for association between PRS of lithium response and TRD.

**Table S3. Association between PRS of lithium response and TRD, excluding TRD cases with lithium use.**

| **PRS** | **Definition** | **Main** | | **Additionally adjust PRS of MDD** | | **Additionally adjust PRS of BIP** | | **Additionally adjust PRS of MDD & BIP** | |
| --- | --- | --- | --- | --- | --- | --- | --- | --- | --- |
|  |  | **OR (95%CI)** | **P (***P_FDR_*) | **OR (95%CI)** | **P (***P_FDR_*) | **OR (95%CI)** | **P (***P_FDR_*) | **OR (95%CI)** | **P (***P_FDR_*) |
| Lithium response | Broad | 1.10  (1.03-1.18) | 0.005 (0.018)* | 1.10  (1.03-1.18) | 0.006 (0.027)* | 1.11  (1.03-1.19) | 0.004 (0.014)* | 1.10  (1.03-1.18) | 0.006 (0.010)* |
| Lithium response | Narrow_1 | 1.11  (1.03-1.20) | 0.009 (0.018)* | 1.11  (1.02-1.20) | 0.014 (0.028)* | 1.12  (1.03-1.21) | 0.007 (0.014)* | 1.11  (1.03-1.20) | 0.010 (0.010)* |
| Lithium response | Narrow_2 | 1.13  (1.04-1.24) | 0.006 (0.018)* | 1.13  (1.03-1.23) | 0.009 (0.027)* | 1.13  (1.04-1.24) | 0.005 (0.014)* | 1.13  (1.03-1.24) | 0.007 (0.010)* |

PRS: polygenic risk score; BIP: bipolar disorder; MDD: major depressive disorder; TRD: treatment-resistant depression.

* FDR<0.05 after correcting for multiple comparisons.

All models were adjusted for the first four PCs.
